# Stress Related Shift Toward Inflammaging in Cosmonauts After Long-Duration Space Flight

**DOI:** 10.3389/fphys.2019.00085

**Published:** 2019-02-19

**Authors:** Judith-Irina Buchheim, Sandra Matzel, Marina Rykova, Galina Vassilieva, Sergey Ponomarev, Igor Nichiporuk, Marion Hörl, Dominique Moser, Katharina Biere, Matthias Feuerecker, Gustav Schelling, Detlef Thieme, Ines Kaufmann, Manfred Thiel, Alexander Choukèr

**Affiliations:** ^1^Laboratory of Translational Research “Stress and Immunity”, Department of Anesthesiology, Hospital of the University of Munich, LMU, Munich, Germany; ^2^Institute of Biomedical Problems, Russian Academy of Sciences, Moscow, Russia; ^3^Institute of Doping Analysis and Sports Biochemistry, Dresden, Germany; ^4^Department of Anesthesiology, Hospital Munich-Neuperlach, Munich, Germany; ^5^Department of Anesthesiology and Surgical Intensive Care Medicine, University Medical Center Mannheim, Medical Faculty Mannheim, Heidelberg University, Mannheim, Germany

**Keywords:** space flight, anandamide, stress, regulatory T cells, CD8^+^ memory T cells, inflammaging

## Abstract

Space flight exerts a specific conglomerate of stressors on humans that can modulate the immune system. The mechanism remains to be elucidated and the consequences for cosmonauts in the long term are unclear. Most of the current research stems from short-term spaceflights as well as pre- and post-flight analyses due to operational limitations. Immune function of 12 cosmonauts participating in a long-duration (>140 days) spaceflight mission was monitored pre-, post-, and on two time-points in-flight. While the classical markers for stress such as cortisol in saliva where not significantly altered, blood concentrations of the endocannabinoid system (ECS) were found to be highly increased in-flight indicating a biological stress response. Moreover, subjects showed a significant rise in white blood cell counts. Neutrophils, monocytes and B cells increased by 50% whereas NK cells dropped by nearly 60% shortly after landing. Analysis of blood smears showed that lymphocyte percentages, though unchanged pre- and post-flight were elevated in-flight. Functional tests on the ground revealed stable cellular glutathione levels, unaltered baseline and stimulated ROS release in neutrophils but an increased shedding of L-selectin post-flight. *In vitro* stimulation of whole blood samples with fungal antigen showed a highly amplified TNF and IL-1β response. Furthermore, a significant reduction in CD4^+^CD25^+^CD27^low^ regulatory T cells was observed post-flight but returned to normal levels after one month. Concomitantly, high in-flight levels of regulatory cytokines TGF-β, IL-10 and IL-1ra dropped rapidly after return to Earth. Finally, we observed a shift in the CD8^+^ T cell repertoire toward CD8^+^ memory cells that lasted even one month after return to Earth.

**Conclusion:** Long-duration spaceflight triggered a sustained stress dependent release of endocannabinoids combined with an aberrant immune activation mimicking features of people at risk for inflammation related diseases. These effects persisted in part 30 days after return to Earth. The currently available repertoire of in-flight testing as well as the post-flight observation periods need to be expanded to tackle the underlying mechanism for and consequences of these immune changes in order to develop corresponding mitigation strategies based on a personalized approach for future interplanetary space explorations.

## Introduction

Long-duration missions to the Moon and Mars are the next ultimate goals for human space exploration. One of the major concerns is the effect of extreme environmental conditions in space, such as microgravity, radiation, confinement, circadian rhythm disruption, physiological as well as psychological stress, on the human immune system. Increased susceptibility to infection in cosmonauts was already reported during the Apollo era, when a surprisingly high incidence of infectious diseases or inflammation related symptoms were reported on board or after spaceflight (Mermel, 2013). Many studies have investigated this issue and data from space analog research has helped greatly to understand single aspects of the multitude of stressors encountered in space (reviewed in Pagel and Chouker (2016)). Today, there is a growing body of evidence showing that the immune system undergoes a variety of changes during and after space travel such as shifts in leukocyte distribution (Crucian et al., 2008, 2013, 2015), monocyte and granulocyte function (Kaur et al., 2004, 2005), changes of cytokine release in plasma and in response to functional stimulation (Crucian et al., 2000, 2014). Most of the available data on the human immune system, however, is based on pre- and post-flight analyses due to procedural limitations or is based on short-term spaceflight missions (Gueguinou et al., 2009; Crucian et al., 2011). There is only limited knowledge of immune alterations that occur during a long-duration mission and the impact of these immune changes after re-adaptation to the conditions on Earth (Crucian et al., 2015). Chronic exposure to stress can exert profound alterations on the immune system. Classical stress hormones like cortisol driven by the hypothalamic-pituitary-adrenal (HPA) axis or the catecholamines norepinephrine and epinephrine have been shown to interfere with immune responses (Sorrells and Sapolsky, 2007). High cortisol levels, again, trigger other stress response systems such as the endocannabinoid system (ECS). Once activated, it helps the human body to adapt to stressful conditions (Hill et al., 2010). Short-term exposure to altered gravity, such as during a parabolic flight, induces the increased release of compounds of the ECS such as anandamide/N-arachidonylethanolamine (AEA) and 2-arachidonoylglycerol (2-AG) in blood (Chouker et al., 2010). Recently, the capability of the ECS to alter immune function is studied intensively and it was shown that endocannabinoids (EC) can exert a strong suppression of innate and adaptive immune responses (Buchheim et al., 2018). The effects of long-term spaceflight on EC blood levels and their participation in the adaptation process aboard are unknown. Interestingly, a constant exposure to a stressful environment together with a constant exposure to antigens and an ongoing inflammatory response can result in a pathogenic phenotype showing features of premature immune aging, with increased cytokine responses and the accumulation of memory and effector T cells (Fagiolo et al., 1992; Franceschi et al., 2000; De Martinis et al., 2005). This so called “inflammaging” is a low-grade, pro-inflammatory state that renders its host at risk for latent viral infections, allergic or autoimmune disease as seen in elderly patients (Ravaglia et al., 2003; Pawelec and Gouttefangeas, 2006; Bauer and Fuente Mde, 2016; Sanada et al., 2018). There are indications that space travel might promote such an age-related inflammatory phenotype prematurely as crew members spend a considerably long time in such an environment and viral shedding and skin exanthema occur frequently in flight (Mehta et al., 2014; Crucian et al., 2016).

As part of the European Space Agency (ESA) and the Russian space agency joint project IMMUNO, a systematic immune function analysis from both innate and adaptive immune responses in 12 cosmonauts was carried out before and after their long-duration space mission (increment duration > 140 days) to the International Space Station (ISS) to gain more insight into immune alterations during the stressful and unique conditions of space. The results from this long-duration spaceflight study increases our current understanding of the functional significance of the immune system changes which occur after long-duration space travels and provide insights in preparation of future exploration class missions.

## Materials and Methods

### Study Design and Ethical Approval

This study was carried out in accordance with the recommendations from the ethical standards of the appropriate institutional and national committees and with the World Medical Association Helsinki Declaration of 1975 (revised in 2008). The protocol was evaluated by the Russian ethical board (Biomedicine Ethics Committee) at the Institute of Biomedical Problems (IBMP), Moscow, and was awarded the protocol number MBI-29 under the Title “IMMUNO.” The protocol was also approved by the ESA medical board and the Russian Space Agency (Roscosmos). All subjects gave written informed consent in accordance with the Declaration of Helsinki. Russian space crew members employed by the Russian Space Agency (ROSCOSMOS) are referred to as “cosmonauts” whereas space travelers employed by NASA or ESA are referred to as “astronauts.” In this manuscript, we will use the term cosmonauts throughout. A total of 12 male cosmonauts (median age 46 years (41/51)) participating in long-term spaceflight missions (median mission duration 162 days (142/181)) were recruited for the IMMUNO study. Sample collection occurred at a maximum of 6 time-points. Sample collection on ground was performed at the Gagarin Cosmonaut Training Centre (GCTC) near Moscow or the DLR training facility in Cologne, Germany. Baseline data collection (BDC) was scheduled 25 days before flight (L-25) and post-flight sampling occurred on day 1 (R+1), day 7 (R+7) and day 30 (R+30) after landing. Two inflight time-points were scheduled for flight day 90 (F+90) and flight day 150 (F+150).

### Stress Questionnaire

The current stress test (CST) is a validated, standardized, self-estimating, one-page paper test that can be completed within 1 min (Chouker et al., 2001). The test is composed of six pairs of contradictory feelings of increasing intensities between which the subject must decide. From the sum of values obtained, the final CST score is calculated, which may range from 1 (no stress) to 6 (maximal stress).

### Cortisol in Saliva

Saliva samples were collected in the morning (7:00–8:00 AM) and in the evening (7:00–8:00 PM) before teeth brushing or food ingestion. Saliva was collected by chewing on a sterile cotton swab for 30–45 s (Salivettes^®^, Sarstedt, Germany) and samples were stored frozen on ground and inflight until further analysis. Free cortisol levels in saliva were quantified with an automated immunoassay system based on the principle of electro-chemiluminescence (Elecsys 2010, Roche, Mannheim, Germany) at the Institute of Clinical Chemistry, Hospital of the University of Munich, Germany.

### Blood Samples

Blood was drawn into blood tubes containing either EDTA or heparin as anticoagulants. The blood was aliquoted into different portions. Three different blood smears per time-point were performed according to standard procedures on ground and inflight. Fresh whole blood samples were transported to Germany on the same day for blood phenotype and immune function analysis while the EDTA anti-coagulated blood sample was centrifuged and immediately frozen on-site at –80°C until further analysis. Aboard the ISS, blood samples were stored at –80°C until download to Earth. Transfer of samples from the landing site to the lab were performed on dry ice.

### Endocannabinoid Concentrations in Blood

Blood samples were quantified for the EC N-arachidonylethanolamine/anandamide (AEA) and 2-arachidonoylglycerol (2-AG) using high-performance liquid chromatography tandem mass spectrometry technique as published elsewhere (Vogeser and Spohrer, 2006; Chouker et al., 2010).

### Differential Blood Count

Absolute white blood cell count and the percentage of each white blood cell type were measured in whole blood samples (Institute of Clinical Chemistry, Hospital of the University of Munich, Germany).

### Surface Adhesion Marker

Polymorphonuclear leukocytes (PMNs) were extracted from whole blood samples through 40 min of separation in Histopaque^®^ (Cat No. 10771, Sigma-Aldrich, Germany) and stained for adhesion molecules β2-integrin (CD18) and L-selectin (CD62L) (all antibodies from BD Heidelberg, Germany) as previously described (Thiel et al., 2001). Data were expressed as MFI.

### Determination of TNF/fMLP-Induced Production of Radical Oxygen Species (ROS)

Isolated PMNs (∼1 × 10^5^) were incubated with dihydrorhodamine 123 (DHR123) (1 μM) (MoBiTec GmbH, Goettingen, Germany) in 0.5 mL HBSS buffer to detect the production of ROS as previously described (Kaufmann et al., 2012). After priming of the cells with TNF (10 ng/mL) for 5 min, PMNs were stimulated with fMLP (10^-7^ M) for another 5 min. As a positive control, cells were stimulated with PMA. Activation of cells was terminated by putting the tubes in ice water. Production of ROS was determined by flow cytometry (BD, Heidelberg, Germany). DHR fluorescence (530 nm) was measured for gated PMN on a FACScan 9235 (Becton Dickinson Immocytometry Systems, Germany) and analyzed through Cell Quest Pro software (BD Biosciences, United States). Data were collected for 5000 events from each sample and are expressed as mean fluorescence intensity (MFI).

### Monocyte Subset Analysis

Monocyte subset analysis was performed using FACS Calibur flow cytometer (Becton Dickinson, United States) with monoclonal antibodies: anti-CD14-FITC, anti-CD14-PE, anti-CD11b-FITC, anti-CD18-PE, anti-CD54-FITC, anti-CD36-FITC, anti-CD16-PE, anti-CD206-PE (IO test, Beckman Coulter, France). 50 μl EDTA whole blood was stained with 5 μl of the relevant antibodies for 20 min at room temperature. In order to reduce Fc-receptor mediated binding of test antibodies, 2.5 μl Human Seroblock reagent (BIO-RAD, United States) were added for 10 min at room temperature. Red blood cells were lysed with 250 μl OptiLyse C Lysing Solution (Beckman Coulter, France) before analysis.

### Immunophenotyping

Blood cells were stained for flow cytometry with 50 μl EDTA whole blood and 5 μl of the relevant antibodies each for 15 min. Red blood cells were lysed with 450 μl BD Lysing Solution (BD 349202, Heidelberg, Germany) before being analyzed by BD Calibur flow cytometer. Live cells were gated based on forward and side scatter profiles. A comprehensive 4-color flow cytometry antibody matrix (BD Multitest^TM^, Heidelberg, Germany) was formulated for peripheral blood immunophenotype analysis. This panel assessed the major leukocyte and lymphocyte subsets, T cell subsets, memory/naïve and central memory T cell subsets, and constitutively activated T cell percentages. For regulatory T (Treg) cells, the following antibodies were used for staining: anti-human CD3 APC, CD4 PerCP, CD25 FITC, CD127 PE (All from BD Biosciences, Heidelberg, Germany). Analysis was performed using BD Multiset and Cellquest Software. For detection of lymphocyte sub-populations, heparinized blood (50 μl) was incubated with antibodies against the cell-surface markers for 10 min at room temperature in the dark to differentiate CD3^+^ (T-lymphocytes), CD3^+^CD4^+^ (T-helper cells), CD3^+^/CD8^+^ (T-suppressor cells/cytotoxic T-cells), CD3^-^CD19^+^ (B-lymphocytes), CD16^+^CD57^+^ (mature natural killer lymphocytes), CD3^+^CD8^+^CD45RA^+^ and CD3^+^CD8^+^CD45RO^+^ naïve/memory CD8+ T-cells, CD3^+^CD4^+^CD45RA^+^ and CD3^+^CD4^+^CD45RO^+^ naïve/memory CD4^+^ T-cells. All antibodies (Multitest 3/8/45/4; Multitest 3/16/+56/45/19; anti-CD3-FITC; anti-CD8-FITC; anti-CD45RA-FITC; anti-CD57-FITC; anti-CD8-PE; anti-CD25-PE; anti-CD28-PE; anti-CD45RO-PE; anti-CD45-Percp; anti-CD3-APC; anti-CD4-APC; anti-CD8-APC) were obtained from BD Life Sciences, Germany. BD FACS lysing solution (BD Lyse; BD Life Sciences, Germany) was added to lyse RBC and incubated for 3 min at room temperature in the dark prior to flow cytometry analysis using a BD FACSCanto System (BD Biosciences; Germany).

### Intracellular Glutathione

Peripheral blood was collected in EDTA- or sodium heparin-containing tubes. PBMCs were isolated by Histopaque^®^ density gradient centrifugation (Sigma-Aldrich, Germany) following the manufacturer’s instructions and were washed once (centrifuged at 235 ×*g* for 15 min) in PBS (Sigma-Aldrich, Germany). Pellets were re-suspended in RPMI-1640 medium, labeled with cell-specific, fluorescence-marked antibodies (CD4^+^ or CD8^+^ lymphocyte subsets were identified by binding of PerCP-conjugated monoclonal antibodies, all BD Heidelberg, Germany) and incubated with the dye Mercury-Orange (Sigma-Aldrich, Germany) for 5 min at room temperature. Intracellular GSH concentrations were determined by flow cytometry using BD FACSCalibur Flow Cytometer.

### Cytokine Release Assay

Heparinized whole blood (500 μl) was supplemented with equal amounts of RPMI Medium and different concentrations of each stimulating antigen (Fungal antigen mixture, *Aspergillus fumigatus* and pokeweed mitogen (PWM)) and incubated for 48 h at 37°C with 5% CO_2_. The PWM-stimulated plasma sample was used as a positive control for whole blood cytokine release. After each time point of incubation, the supernatants were collected and stored at –20°C until further analysis. The supernatants were then analyzed simultaneously for 27 cytokines (Bio-Plex Pro Human Cytokine Assay; Bio-Rad Laboratories, Germany), including IL-1β, IL-1ra, IL-2, IL-6, IFN-γ, TNF in accordance with the manufacturer’s instructions. The samples were analyzed using a Bio-Plex 200 instrument with Bio-Plex BioManager analysis software. The concentrations of cytokines were measured by comparing the bead color of the bound antibodies and the MFI from each set of beads against a verified standard curve.

### Statistics

Statistical evaluation and plotting of the data set was done using SPSS 24 (IBM, United States) and SigmaPlot 12 (Systat Software Inc., United States). Data was tested for normal distribution using the Kolmogorov-Smirnov Test. Box-Cox transformation of the data was performed only when residuals of the tested variable were found to be non-normally distributed. The data was then analyzed using a linear mixed effects (LME) model, where the time-points measured were regarded as fixed and subjects as random effects. A value of *P* < 0.05 was considered as statistically significant.

## Results

### Analysis of Stress Through Self-Evaluation Questionnaire and Biochemical Markers

**FIGURE 1:**
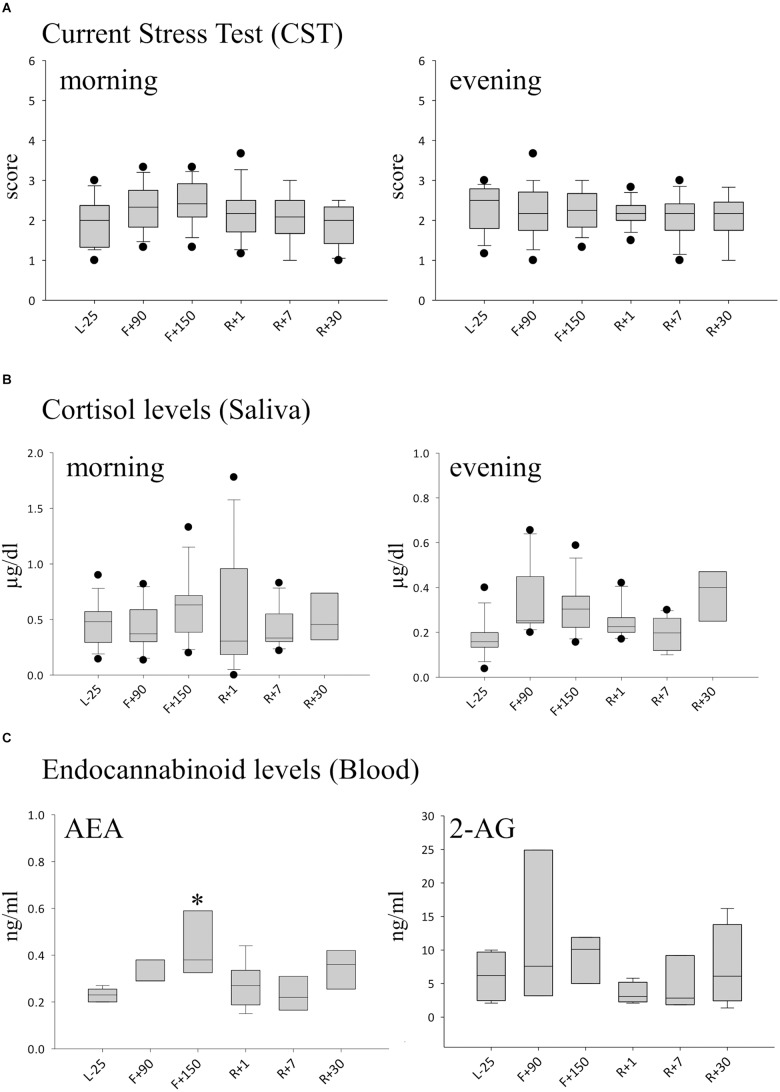
Markers of stress during long-term spaceflight. **(A)** Scores calculated with the CST questionnaire in the morning (left panel) and evening (right panel), *n* = 12 **(B)** Cortisol in saliva measured in the morning (left panel) and evening (right panel), *n* = 8 **(C)** Endocannabinoid levels of AEA (left panel) and 2-AG (right panel) in blood, *n* = 5, data is expressed as median ± 95th and 5th percentile, ^∗^ = *P* < 0.05 vs. other timepoints.

To investigate whether exposure to the special environment aboard the ISS is perceived as stressful by cosmonauts, we used subjective and objective test methods. The subjective stress level was evaluated in the morning and evening using the CST questionnaire. The mean calculated preflight scores were 2.01 (±0.66) in the morning and 2.39 (±0.63) in the evening (Figure 1A). Over the time-course of the mission, the values moderately increased but did not significantly differ between daytime or mission time-points, indicating a personal perception of low stress during the mission. Similar results were obtained when we measured cortisol in saliva samples. Circadian differences with slightly higher levels in the morning and lower levels in the evening were observed, however, no significant changes were detected over time. The evening values showed a slight increase of cortisol levels on F+90 but without statistical significance (Figure 1B). The blood concentrations of the endocannabinoids N-arachidonylethanolamine/anandamide (AEA) and 2-arachidonoylglycerol (2-AG) were measured as another objective stress parameter. AEA was found to be significantly increased at the second inflight time point (F+150) compared to ground (Figure 1C).

### Alterations of Peripheral Blood Leukocyte Phenotypes After Landing

**FIGURE 2:**
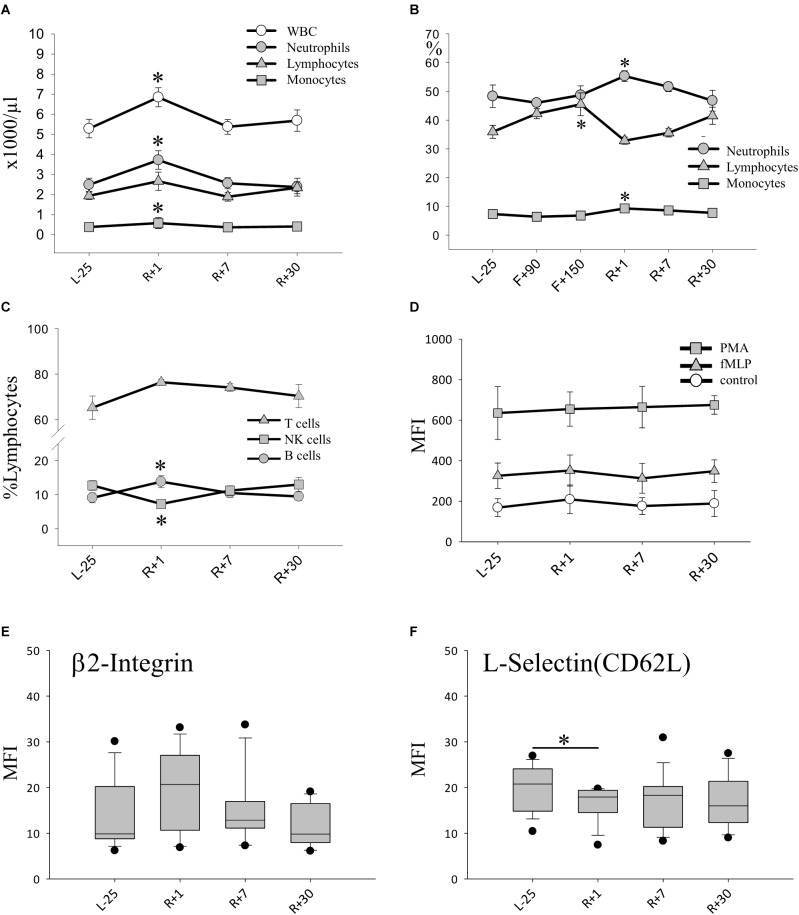
Leukocyte phenotype after long-duration space flight. **(A)** Absolute count of leukocytes (WBC) and subsets pre and post-flight, data expressed as mean ± SEM, *n* = 11 ^∗^ = *P* < 0.05 vs. L-25 control **(B)** Relative counts (%) of neutrophils lymphocytes and monocytes evidenced by blood smear analysis, data expressed as mean ± SEM, *n* = 8 on F+90 and *n* = 10 on F+150 ^∗^ = *P* < 0.05 vs. L-25 control **(C)** Relative counts (% lymphocytes) of lymphocyte subsets pre and post-flight, Data expressed as mean ± SEM, *n* = 12 ^∗^ = *P* < 0.05 vs. L-25 control **(D)** Expression of adhesion molecule β2 integrin (CD18) on the surface of PMNs. Data is expressed as median ± 95th and 5th percentile **(E)** Expression of adhesion molecule L-selectin (CD62L) on the surface of PMNs, data is expressed as median ± 95th and 5th percentile, *n* = 12, ^∗^ = *P* < 0.05. **(F)** Production of H_2_O_2_ measured after stimulation with PMA (positive control), with TNF-α/fMLP (physiologic stimulus) or without stimulus (Control), data expressed as mean ± SEM, *n* = 12.

Next, we wanted to evaluate the effect of long-term spaceflight on leukocyte populations. The total number of leukocytes (WBC) was significantly increased immediately after landing (R+1). Compared with baseline, neutrophil and monocyte numbers increased by 50% at this time-point. Absolute numbers of lymphocytes remained constant pre- and post-flight (Figure 2A). Due to procedural limitations, we were able to study leukocyte changes inflight only by analyzing blood smears generated percentages. The relative counts obtained showed an increase of neutrophils and monocytes post-flight, confirming the results obtained based on absolute numbers. When we analyzed the monocyte population further on ground, we found a significant decrease of CD14^+^CD16^+^on R+1. On R+7 this decrease was still observed but did not reach statistical significance due to high inter-individual variations (data not shown). Interestingly, the relative lymphocyte population inflight was significantly increased and returned to normal numbers post-flight (Figure 2B). When studying the lymphocyte subgroups after return in whole blood samples, an increase in B-cells was seen, whereas T cells showed no alteration. In particular, natural killer (NK) cells dropped by 60% post-flight (Figure 2C).

### Activation of Polymorphonuclear Leukocytes (PMN) After Spaceflight

Since neutrophil counts rose in relative and absolute terms on R+1, we turned to look at their state of activation and function. The capability to produce reactive oxygen species (ROS) was tested, which remained stable after spaceflight in response to a strong (PMA) or physiologic stimulus fMLP/TNF (Figure 2D). Similar results were obtained when we analyzed the amount of cellular antioxidant glutathione which was unaltered in all cell types over all time-points (data not shown.) Furthermore, the expression of adhesion molecules β2-integrin (CD18) and L-selectin (CD62L) on the surface of PMN was characterized. The expression of β2-integrin was already elevated preflight and remained so until 30 days post-flight without significant changes (Figure 2E). On the other hand, a notable decrease in cell-surface-bound L-selectin molecules was detected upon return (R+1), indicating an increased shedding of L-selectin and thus strong activation of PMNs (Figure 2F).

### Amplified TNF Response to an *in vitro* Exposure to Fungal Antigen

**FIGURE 3:**
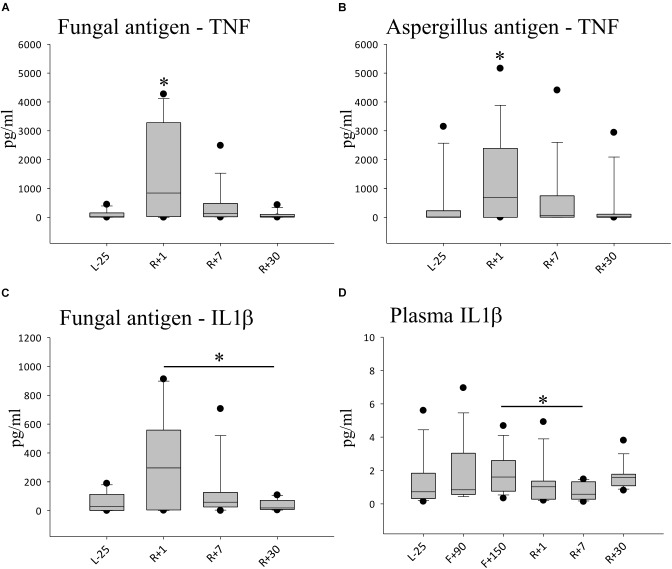
Cytokine profiles against simulated fungal infection. **(A)** Quantification of TNFα after 48 h of stimulation with fungal antigen, data is expressed as median ± 95th and 5th percentile, *n* = 12 ^∗^ = *P* < 0.05 vs. other timepoints **(B)** Quantification of TNFα after 48 h of stimulation with *Aspergillus* antigen, data expressed as median ± 95th and 5th percentile, *n* = 11,^∗^ = *P* < 0.05 vs. L-25 control **(C)** Quantification of IL-1β after 48 h of stimulation with fungal antigen, data is expressed as median ± 95th and 5th percentile, *n* = 5 ^∗^ = *P* < 0.05 **(D)** Quantification of IL-1β in Plasma, data is expressed as median ± 95th and 5th percentile, *n* = 9 on F+90, *n* = 11 on F+150, ^∗^ = *P* < 0.05.

To analyze the overall cytokine immune response to an infection, we challenged whole blood samples with distinct antigens for 48 h. The production of pro-inflammatory cytokines, TNF, IL-2, IL-6, IFN-γ as well as IL-1β and IL-1ra, were monitored in culture supernatants in the presence of fungal antigen mixture or *Aspergillus* antigen (Figure 3). At R+1, the secretion of TNF was highly and significantly increased compared to all other time-points in response to fungal antigens, TNF production increased by 10-fold (Figure 3A). At time point R+7, we could still observe elevated levels of TNF, though it was not statistically significant. Similar results were obtained for *Aspergillus* antigen (Figure 3B). IL-1β release was also markedly increased after stimulation with fungal antigens. Comparing these results with the measured cytokine plasma concentrations, IL-1β was significantly increased in flight but returned to baseline concentrations at R+1 and could therefore not account for the higher amounts detected after stimulation. For IL-2, IL-6 and IFN-γ responses, no significant difference from baseline was detected. In non-challenged control samples, no alteration of TNF, IL-2 and IFN-γ levels were measurable throughout the study interval (data not shown).

### Regulatory T-Cell Subsets and Cytokine Profiles Display a Reduction of the Anti-inflammatory Capacity

**FIGURE 4:**
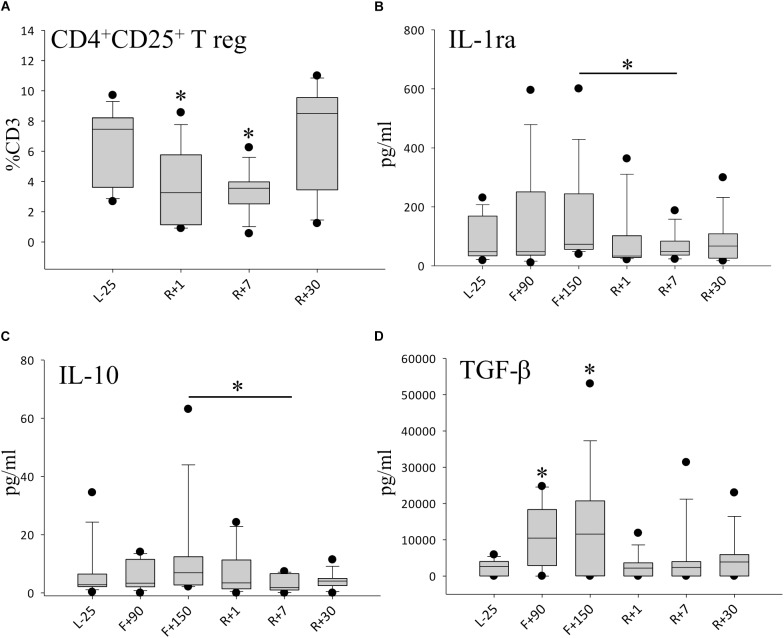
Anti-inflammatory milieu after spaceflight. **(A)** Percentage of CD4^+^CD25^+^T regulatory cells (Treg) in peripheral blood, *n* = 12. **(B)** Quantification of IL-1ra in plasma, *n* = 9 on F+90, *n* = 11 on F+150 **(C)** Quantification of IL-10 in plasma, *n* = 9 on F+90, *n* = 11 on F+150, **(D)** Quantification of TGF-ß in plasma, *n* = 8 on F+90, *n* = 10 on F+150; all panels: data is expressed as median ± 95th and 5th percentile, ^∗^ = *P* < 0.05 vs. L-25 control.

Since we observed higher leukocyte numbers and a cytokine response overshoot following space flight, we looked further into the T cell population. Among the T cell subsets characterized, no significant difference was detected for CD4^+^ memory cells and CD4^+^ naïve cells (data not shown). Interestingly, anti-inflammatory regulating T cells (Tregs) (CD4^+^CD25^+^CD127^low^) were significantly reduced by nearly 30% after spaceflight on R+7 (Figure 4A). Anti-inflammatory cytokine IL-1ra (Figure 4B) as well as regulatory cytokines IL-10 (Figure 4C) and TGF-β (Figure 4D) were found to be reduced after spaceflight but highly increased inflight, suggesting a pro-inflammatory immune status with a concomitant reduction in the anti-inflammatory capacity.

### Repertoire Shift of CD8^+^ T Cells

**FIGURE 5:**
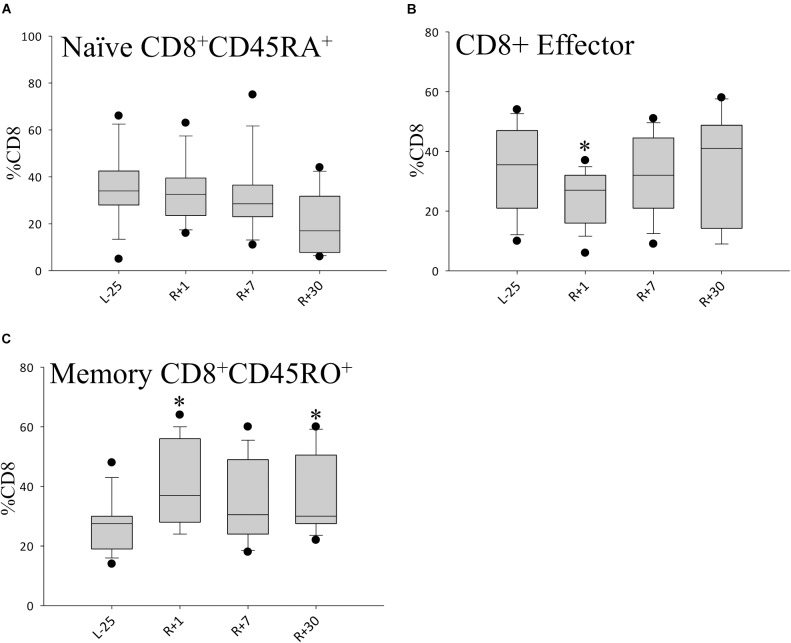
Shifts in CD8+ cell population. Bar diagrams showing percentages of CD8^+^ naïve **(A)** CD8^+^ effector **(B)** and CD8^+^ memory **(C)** T cell populations. Data is expressed as median ± 95th and 5th percentile, *n* = 12 in all panels, ^∗^ = *P* < 0.05 vs. L-25 control.

When looking at the CD8^+^ T cell compartment, we found that CD8^+^ naïve cells remained largely unaltered by spaceflight (Figure 5A), while CD8^+^ effector T cells were found to be transiently diminished directly after return (Figure 5B) but reverted to normal values on day 7. Additionally, we observed an increase in the CD8^+^ memory cell repertoire. This shift persisted until day 30 after return, the last time-point of the study, indicating a prolonged change in the CD8^+^ T cell compartment. Values at R+7 did not reach statistical significance due to interindividual differences (Figure 5C).

## Discussion

This study aimed to assess immune function in ISS crew members before and after long-duration spaceflight by evaluating stress levels and through performing a variety of analyses on both the innate and adaptive immune systems. Our study revealed a general activation of stress response systems as evidenced by high levels of AEA inflight. We detected a pro-inflammatory state characterized by an aberrant peripheral blood leukocyte distribution, elevated neutrophil activity and highly amplified TNF and IL-1β response against fungal antigen stimulation. Moreover, we observed a reduction in anti-inflammatory capacities since regulatory T cells, as well as regulatory cytokines IL-1ra, IL-10 and TGF-β, were found to be reduced post-flight. Finally, a shift in the CD8^+^ cell repertoire with elevated CD8^+^ memory T-cell counts that persisted 30 days post-flight argues for a prolonged alteration in adaptive immunity.

### Stress Response Systems in Chronic Stress

Stress can be regarded as the reaction of an organism to changes in the surrounding environment (Selye, 1956). An acute stress response, the so called “fight or flight” reaction, leads to the redistribution of blood flow to distinct organs in order to ensure survival in case of a life threatening event (Cannon, 1929). The hypothalamus directs the fast response (in seconds) via the sympathetic nerve system, where the medulla of the adrenal glands release catecholamines (epinephrine and norepinephrine), and concurrently a slower response via the HPA axis, resulting in a release of cortisol. In this study, we asked cosmonauts about their personal experiences of stress using the CST questionnaire and they did not report acute strain. Cortisol levels were moderately affected and are in line with the CST results. The endocannabinoid AEA, however, was highly increased inflight, demonstrating a biological stress response to the environment aboard the ISS. The ECS is an important stress response system and has multiple roles in a myriad of physiological processes of stress (Chouker et al., 2010; Dlugos et al., 2012; Hauer et al., 2013; Neumeister et al., 2015), metabolism (Campolongo et al., 2009), sleep and activity patterns (Richard et al., 2009; Feuerecker et al., 2012). Most importantly, endocannabinoids are potent immune modulators (Sardinha et al., 2014; Buchheim et al., 2018). They act via the endocannabinoid receptors (CB) 1 and 2, which are not only expressed in the central and peripheral nervous systems (CB1), but also on the surface of many leukocytes (CB2). Our data suggest that AEA is a good measure for chronic stress and does not necessarily translate into psychological burden as seen in the low self-evaluation questionnaire (CST) scores. After an event causing acute stress such as landing on Earth (R+1), EC levels remained low. It appears that EC release is stimulated by conditions in space and not by the acute stress exerted during landing, which is well in line with the knowledge that acute and chronic stresses exert very different effects on the immune system (Moynihan, 2003). Correspondingly, our data show similarities to previously reported results, where the ECS was responsible for the modulation of constant baseline ROS release (without stimulus) in neutrophils and did not alter ROS production after stimulation with fMLP and TNF (Buchheim et al., 2018).

### Leukocyte Recruitment Triggered by an Acute Stressor

The immune system reacts to acute stress by releasing leukocytes (Meehan et al., 1993; Stowe et al., 2003; Dhabhar et al., 2012). Neutrophils belong to the first line of innate immune defense and are the first to arrive at sites of infection. Functional defects of neutrophils are characterized by poor wound healing and recurring infections. In this study, we observed neutrophil activation following spaceflight (R+1) characterized by a decrease in L-selectin CD62L expression on the cell surface of neutrophils. Neutrophils are tasked with the production of cytotoxic granules containing ROS and their release during oxidative burst (Bian et al., 2012; Liu et al., 2012; Kolaczkowska and Kubes, 2013). ROS release remained stable and antioxidative levels of glutathione were preserved under physiologic stimuli. It would be of great interest to examine ROS release under in-flight conditions together with assessing AEA levels, since the ECS plays a decisive role in controlling oxidative stress and thus in maintaining cellular homeostasis (Lipina and Hundal, 2016). Due to procedural limitations, this is currently not possible in space. One might speculate that the high levels of AEA could be responsible for the stable ROS production observed over the course of this study. Previous investigations following short-duration spaceflight also reported changes of neutrophil function demonstrated by enhanced chemotactic activity after landing, increased neutrophil adhesion to endothelial cells and significantly changed L-selectin expression (Stowe et al., 1999). Interestingly, L-selectin expression on neutrophils was significantly increased after short-duration spaceflight (Stowe et al., 1999), in contrast to our findings following long-duration spaceflight. This difference may result from the cumulative effects of long-duration mission related factors (i.e., microgravity, radiation, or re-adaptation to earth environment) on neutrophil activation. The observed changes in the peripheral blood leukocyte distribution post-flight (R+1) are largely consistent with previous reports on immune changes following short-term flights (Crucian et al., 2008) and a pilot study after long-term spaceflight (Crucian et al., 2015). Directly following landing, we observed elevated leukocyte counts including both neutrophils and monocytes, as well as B-cells. This phenomenon may at least in part be triggered by the landing process, which induces dramatic acute physical strain on the human body owing to the combination of microgravity, hyper-gravity and fierce vibrations.

### Hypersensitivity Toward Fungi and Proinflammatory Phenotype

The percentual increase in lymphocytes in blood smears inflight indicate that the adaptive immune system was also activated in long missions and not only just a component of the innate immunity as previously reported (Crucian et al., 2015). Since fungal colonization of the ISS is a known burden (Novikova et al., 2006), it is particularly interesting to understand the interactions of stress, fungal antigen load aboard and the resulting immune response in the long term. When we challenged the immune system with fungal antigens upon return from long-term spaceflight, an overshoot of immune response with pro-inflammatory cytokines TNF and IL-1β which continued to be elevated 7 days after return to Earth. Moreover, IL-1β levels were significantly elevated in flight but dropped rapidly post-flight, indicating that the measured overshoot response was not biased simply due to higher plasma concentrations of the cytokine. Previously, we have shown that under ECS activation the fungal antigen response is abrogated (Buchheim et al., 2018). Here, we observe that plasma cytokine concentrations do not necessarily correlate with the cytokine response after antigen stimulation. Additionally, we observed a significant reduction in NK cells at R+1. NK cells are critical in the defense against fungal infections and conversely, fungi can exert immunosuppressive effects on NK cells (Schmidt et al., 2017). It was reported that NK cell cytotoxicity was reduced after short-term shuttle missions without a drop in absolute numbers (Mehta et al., 2001).

In accordance with the boosted response to fungal antigens, we observed a reduction of Treg cells upon return to Earth, suggesting a compromised function in suppressing T cell proliferation and pro-inflammatory cytokine secretion. Many investigations have demonstrated that Treg cells are capable of down-regulating antigen-specific T-cell responses (Dejaco et al., 2006). The changes in anti-inflammatory plasma cytokine levels here mirror a Treg suppressed state. We also observed a significant increase in the regulatory cytokines IL-1ra and IL-10 inflight which dropped rapidly after return. The changes in concentrations of TGF-β were even more striking. This reflects the important role TGF-β plays in the function of Tregs and is thought to be critical for the maintenance of tolerance in the immune system (Ouyang et al., 2010; Kehrl et al., 2014). Concomitantly, we observed a shift of cells toward CD8^+^ memory T cells, which persisted until the end of the study, whereas the CD4^+^ T cell naïve and memory populations remained largely unaltered. It has been shown that CD8^+^ T cells are more unstable and increase in the immune aging phenotype (Goronzy et al., 2007). This does not necessarily refer to chronological age but rather, is an expression of the imbalance of pro-and anti-inflammatory mechanisms (Ventura et al., 2017). Such alterations, should they persist, could lead to diseases associated with immune imbalance including chronic inflammation, autoimmune or other inflammation related diseases (Franceschi et al., 2007).

### Immune Response Shift in Chronic Stress Can Trigger Inflammaging

The effects of spaceflight on the immune system have two aspects: acute versus chronic stress and inflight versus post-flight. A strong acute stress reaction to a potentially life-threatening event such as the return to Earth serves as an immune activator that mobilizes immune cells and activates neutrophils regardless of the overall immune status. Chronic exposure to stress leads to an activation of similar mechanisms over a longer period of time. In the acute stress response, T cells are redistributed to areas of actions such as the blood stream or the skin, because skin damage is a likely event during a fight or flight situation. If, however, this alarm signal is maintained over a longer period of time, fewer T cells will respond to the recruitment leading to a reduced number of T cells in the skin, which has been shown in delayed type hypersensitivity (DTH) skin tests during spaceflight (Dhabhar and McEwen, 1997). Others have also noted a reduction in T cell proliferation and activity in the skin (Chang et al., 2012; Sonnenfeld, 2012). Similarly, delayed mucosal wound healing and a decrease in the key cytokine IL-1β can be observed during chronic stress (Marucha et al., 1998). A typical pattern in chronic stress is a shift to a T helper cell type 2 response through the decrease in pro-inflammatory cytokines (TNF, IL-1β, IL-6, INF-γ) and higher levels of anti-inflammatory cytokines (IL-10, TGF-β) (Marshall et al., 1998; Kang and Fox, 2001). These shifts were also observed in our study inflight, showing that cosmonauts were exposed to stress although they might not have perceived it as such. High levels of AEA inflight indicate a form of adaptation to this environment and the body’s efforts in maintaining cellular homeostasis. If we had been able to conduct a stimulated antigen response also inflight, a reduction of cytokines after antigen stimulation would be expected (Buchheim et al., 2018). The technical limitations aboard the ISS currently prevent us from carrying out these tests in space. Prolonged exposure to this very specific environment does not only lead to immunological adaptation but triggers shifts in cellular compartments of the immune system. The data on Tregs and CD8^+^ cells together with the functional tests suggest a special conglomerate of stressors inflight, triggering a hyperinflammatory and aging immune phenotype (inflammaging) that persists even after 1 month after the return to Earth. It will be valuable to prolong the post-flight observation period for long-term effects such as shifts in the T cell repertoire. This could identify crew personnel at risk for developing such diseases and encourage appropriate preventive measures as well as further monitoring.

In summary, the results of this study indicate long-duration spaceflight could trigger inflammaging, which may expose cosmonauts to risks for hypersensitivity diseases such as allergies or autoimmune diseases. Further on-board immune functional immune testing and a longer post-flight observation period are necessary to understand the underlying mechanism and the consequences for cosmonaut health in the long-term. It is then necessary to develop corresponding mitigation strategies based on a personalized approach for interplanetary space explorations.

## Author Contributions

AC, MT, MF, IK, DT, and GS designed the study. SM, MR, GV, IN, and SP performed and supported the data sampling. SM, MH, KB, and DM performed the sample analysis. J-IB performed data analysis. J-IB and AC drafted the manuscript.

## Conflict of Interest Statement

The authors declare that the research was conducted in the absence of any commercial or financial relationships that could be construed as a potential conflict of interest.
